# Sensor-Based Solid Waste Handling Systems: A Survey

**DOI:** 10.3390/s22062340

**Published:** 2022-03-18

**Authors:** S. Vishnu, S. R. Jino Ramson, M. S. S. Rukmini, Adnan M. Abu-Mahfouz

**Affiliations:** 1 Department of Electronics and Communication Engineering, Vignan’s Foundation for Science, Technology and Research, Guntur 522213, India; sv_ece@vignan.ac.in (S.V.); drmssr_ece@vignan.ac.in (M.S.S.R.); 2 School of Electrical and Electronics Engineering, VIT Bhopal University, Bhopal 466114, India; 3 Council for Scientific and Industrial Research (CSIR), Pretoria 0184, South Africa; a.abumahfouz@ieee.org; 4 Department of Electrical and Electronic Engineering Science, University of Johannesburg, Johannesburg 0001, South Africa

**Keywords:** Internet of Things, solid waste management, trash bin, LoRaWAN, Wi-Fi, smart city, remote monitoring

## Abstract

As a consequence of swiftly growing populations in the urban areas, larger quantities of solid waste also form rapidly. Since urban local bodies are found to be unable to manage this perilous situation effectively, there is a high probability of risks relative to the environment and public health. A sudden change is indispensable in the existing systems that are developed for the collection, transportation, and disposal of solid waste, which are entangled in turmoil. However, Smart sensors and wireless technology enable cyber-physical systems to automate solid waste management, which will revolutionize the industry. This work presents a comprehensive study on the evolution of automation approaches in solid waste management systems. This study is enhanced by dissecting the available literature in solid waste management with Radio Frequency Identification (RFID), Wireless Sensor Networks (WSN), and Internet of Things (IoT)-based approaches and analyzing each category with a typical architecture, respectively. In addition, various communication technologies adopted in the aforementioned categories are critically analyzed to identify the best choice for the deployment of trash bins. From the survey, it is inferred that IoT-based systems are superior to other design approaches, and LoRaWAN is identified as the preferred communication protocol for the automation of solid waste handling systems in urban areas. Furthermore, the critical open research issues on state-of-the-art solid waste handling systems are identified and future directions to address the same topic are suggested.

## 1. Introduction

Everyday, the generation of waste increases and is paving the way for serious negative environmental impacts all over the world [1]. It is incontrovertible that all human activities end up generating a considerable quantity of waste in all forms of matter [2]. There is a huge impact on our environment due to undesigned modernization along with industrialization, which leads to a sudden increase in solid waste, nuclear waste, and other perilous wastes [3].

**Figure 1:**
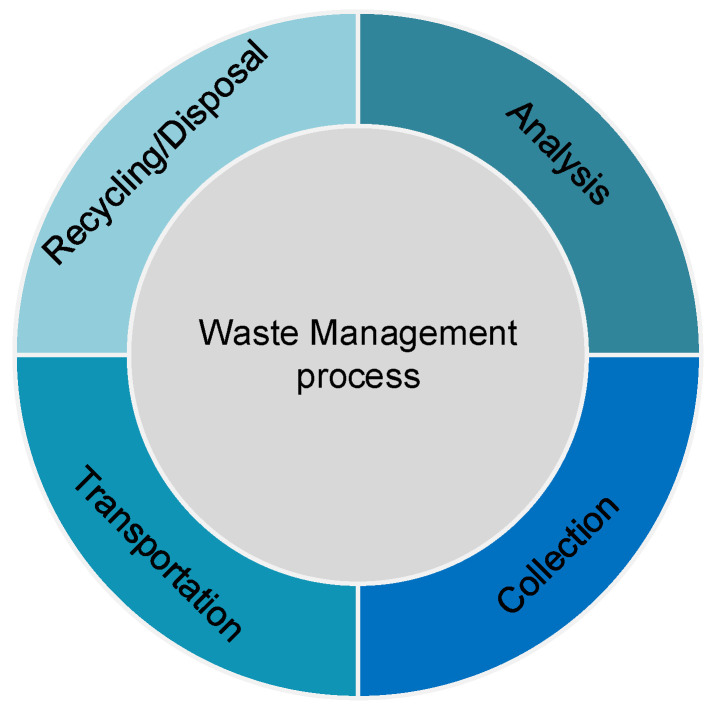
Process in solid waste management.

Waste management refers to the reduction in the amount of waste being produced and the disposal of the same products by using adequate measures [4,5]. Waste may come in many shapes and forms, including solid waste being produced from domestic sources, infectious waste produced from hospitals, and radioactive waste produced by nuclear reactors [6,7]. Waste management includes the collection, transportation, recycling or disposal, and analysis of waste materials, as shown in Figure 1. Moreover, waste management is relatively new as an industry, which is flourishing day-by-day because of its extreme impact on the ecosystem.

**Figure 2:**
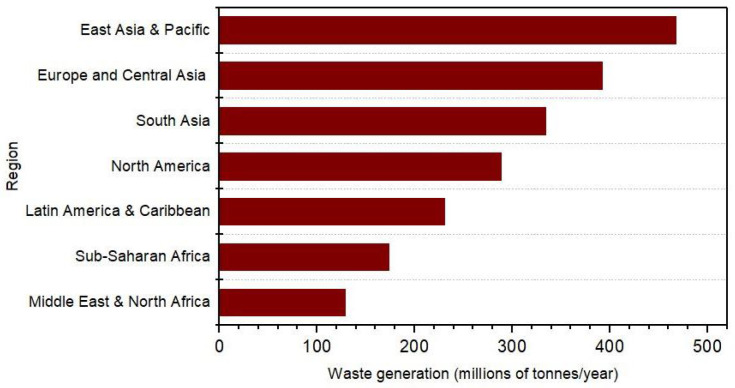
Projected waste production by region (millions of tonnes/year).

Amusingly, the rate of waste produced is linked to towering economic growth, and it is really an attribute of advanced countries. However, the quantity of waste generated depends on the region, season, and many other factors, which show the difficulty in proper waste disposal. The world bank statistics [8] on the projected regional waste production in millions of tonnes are shown in Figure 2.

The management of waste is a complex multi-level process that involves government policy making, legal, financial, administrative, and research facilities [9]. The high cost associated with the safe and efficient management of waste makes investing in this industry difficult [10]. Therefore, a lot of research and development in this sector is required to establish efficient waste management systems [11]. The conventional waste management process begins with the disposal of waste in trash bins near the place of origination. At predetermined times, municipal or private enterprise vehicles pick up waste and move it to temporary collection units. However, these approaches may result in improper waste management due to the following circumstances.

The collection of solid waste on a daily basis constitutes a loss of time, fuel, and labor in cases where trash bins are empty. Alternatively, the collection of solid waste once a week may lead to a risk of overflowing the trash bins. Consequently, solid waste spreads across the region, causing possible heath problems and environmental pollution. Moreover, when cities are large in size, an effective solid waste management system is of great importance. However, the technological developments in the field of sensors and wireless communications pave the way to automate solid waste management systems. Several automation techniques are available that begin with RFID technology relative to the latest Internet of Things (IoT)-based models. Nevertheless, for an efficient solid waste management system, a challenging task is to determine the appropriate mix of sensor and communication technologies with optimum solutions.

Few surveys and study articles on solid waste management systems were reported previously. Most of them emphasized the trend at the time or on a wider range of sensor and communication technologies that aided the design of solid waste management systems. A survey on environmental informatics applied to solid waste management is presented in [12]. The limitations and drawbacks of various waste management models are highlighted in this work. Moreover, it presents a timeline of various technologies applied to the development of a solid waste management system. However, it does not discuss advanced communication technologies such as low power wide area networks (LPWAN), which is one of the enabling technologies of IoT. In [13], a survey is performed to analyze the existing information and communication technologies (ICTs) and their use in solid waste management systems. It discussed the concerns and obstacles associated with implementing an ICT-enabled system. However, the latest technological approaches towards solid waste management systems are not included in this study. A systematic review of various technologies and decision support systems for the efficient handling of solid waste is presented in [14]. This work emphasizes much on decision support systems and does not provide a critical comparison of advanced ICT technologies adopted for the design of solid waste handling systems. A study on IoT frameworks for waste management in smart cities is presented in [15]. It compares various IoT reference models from the literature and highlights the research issues and challenges among them. However, the study does not analyze the identified solutions in terms of power consumption and communication range. An analysis of various ICT technologies, especially IoT and blockchain-oriented approaches towards the design and development of solid waste management systems, is described in [16]. It highlights the limitations of wireless communication technologies adopted for IoT architectures. However, from the analysis of previous studies and reviews on solid waste management systems, it is evident that none of the existing works presented a hierarchical approach on how evolution in communication technology revolutionized the solid waste management industry. Moreover, none of the existing works distinguish the WSN and IoT-based approaches for solid waste management systems.

The main contributions of this study are as follows:

- A comprehensive analysis of RFID, WSN, and IoT-based approaches towards the automation of solid waste handling systems.
- Each category is analyzed with a typical system architecture. Significance and limitations for the same are discussed.
- Recommends apt communication technology for IoT-based solid waste handling systems.
- The critical open research issues on state-of-the-art solid waste handling systems are concluded from the review and recommendations for future directions are presented.

The remainder of this article is organized as follows. Section 2 describes the methodology adopted for the survey. Section 3 presents RFID-based approaches for solid waste management systems. Section 4 describes various WSN-based approaches towards solid waste management systems. Section 5 presents various IoT-enabled approaches towards solid waste management systems. Section 6 presents the comparison and discussions on the various approaches in solid waste management. Section 7 describes research gaps and future directions for solid waste management. Finally, the article is concluded in Section 8.

## 2. Methodology

The main focus of this survey is to analyze the evolution of automation approaches towards solid waste handling systems. “Communication technologies for solid waste handling system” is framed as the research question. Research articles have been collected from various databases such as ACM Digital Library, IEEE Xplore, ScienceDirect, and SpringerLink. The publications from 2007 to 2021 are considered for this survey. To obtain quality publications from the pool of publications, the following strategy is adopted.

- Similar approaches published in different years are excluded from the survey.
- Conference papers with proof of concept are only included for the survey.

**Figure 3:**
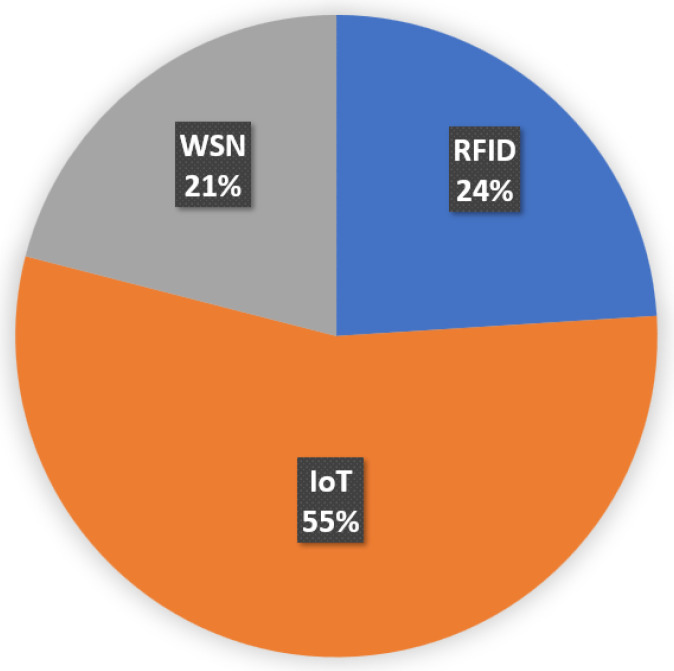
Distribution of publications based on technologies.

The publications considered for the survey are classified according to the evolution of technology such as RFID, WSN, and IoT. The distribution of publications based on the aforementioned categories are shown in Figure 3. Among the publications 55% belongs to IoT-based approaches, 21% belongs to WSN-based approaches and 24% belongs to RFID-based approaches towards the solid waste handling systems. Furthermore, the significance, challenges, and limitations of the aforementioned categories are critically analyzed to obtain open research issues and possible future research directions.

## 3. RFID Based Solid Waste Management Systems

RFID technology is an automated identifying system that is primarily based on RF microwave transmission and is seen as a natural extension of previous identification systems such as bar codes, magnetic cards, and smart cards [17]. Despite the fact that the RFID system’s theory of operation was conceived at the beginning of the radio frequency communications era, its use is steadily increasing today [18]. RFID is a technology that allows users to read information from tags and communicate it to an information-processing system over a radio frequency range without the need for a physical connection. An antenna, a tag, and a reader are the three parts of an RFID system. The antenna transmits a signal that activates the transponder using radiofrequency waves. When the tag is engaged, it sends data back to the antenna. The transmission ranges of low-frequency RFID systems are short, while those of high-frequency RFID systems are longer. Contactless payments [19], automobile [20], library [21], retail supply chain management [22], pharmaceutical [23], industry [24], ticketing [25], and solid waste management systems [26] are only a few of the applications where RFID technology is used.

The main objectives of RFID based solid waste management systems are as follows:

- To obtain information on the waste collection area and the respective collecting time;
- To develop a system for monitoring and tracking of waste collection trucks and waste bins;
- To obtain information on the quantity of solid waste inside the bin and the surroundings.

### 3.1. System Architecture

**Figure 4:**
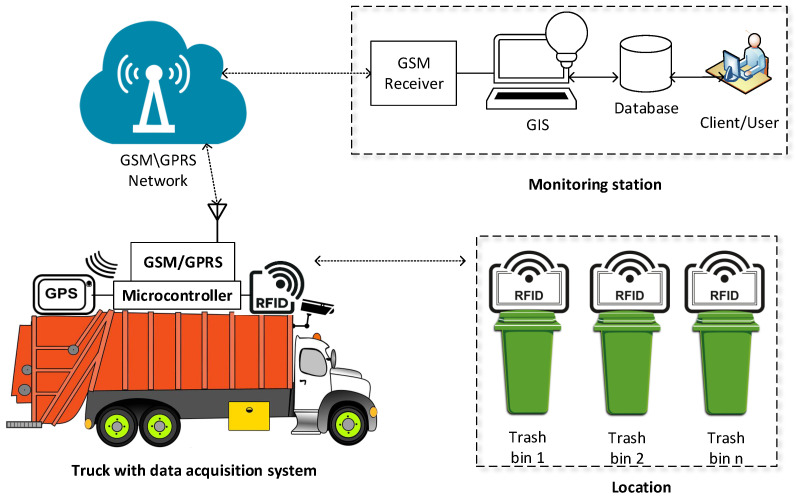
A typical system architecture for RFID-based Solid Waste Handling System.

A typical system architecture of the RFID-based solid waste handling system is shown in Figure 4. Communication technologies such as general packet radio service (GPRS), global positioning system (GPS), radio frequency identification (RFID), and geographic information system (GIS) including camera have been integrated for developing the system [27].

An RFID tag is attached to all the bins to provide unique identity. The truck, which is used to collect waste from the bins, is mounted with a data acquisition system comprising an RFID reader, camera, GPS, and GSM Module. Once the truck approaches the bin area, the RFID reader detects the RFID tag installed on the bin, the GPS module obtains the real-time location, and the camera captures images prior to and subsequent to waste collection in order to determine the waste in the bin and its surroundings. The data from the truck’s data acquisition system are recorded and sent to a central monitoring station by using a GSM or GPRS network. The monitoring station comprises a receiver, GIS, database and a monitoring terminal for providing user interface. The GIS and database management systems are responsible for mapping of truck position and bin location to optimize truck routes according to waste estimation.

#### RFID-Enabled Trash Bin Level Monitoring Systems

Numerous trash bin level monitoring systems have been developed using RFID technology. Table 1 shows a summary of RFID enabled trash bin level monitoring system. The work presented in [27,28] is a purely RFID-based system, which is not using any sensors for bin level measurement. The camera-equipped on the truck is capturing images of the trash bins, which will be sent to the central monitoring station for verifying the waste collection process. Moreover, the system is not using any routing methods to optimize waste collection. The system presented in [29] used an additional infrared sensor for measuring the trash bin’s filled level. This system is not using any camera, since sensors are equipped to assess the emptiness of trash bins. In [30], a series of sensors was used to assess the unoccupied level and pressure of trash bins. A camera is also equipped in the system for the establishment of GIS for assisting the truck driver to execute optimized waste collection from the trash bins. In [31], an outdoor trash bin monitoring system based on RFID is presented. This system used a load-cell sensor to measure the weight of the trash bins. Threshold levels are set according to the filled and empty trash bin’s weight.

The RFID-based system presented in [32] is integrated with photoelectric and image sensors to obtain the filled status of the trash bins. A GPS module is also equipped to obtain the accurate locations of the bins to define optimized routing for the waste collection truck. An RFID-based smart trash bin is proposed in [33]. This system performs waste material classification with a web-based information system running on the remote server. The trash bins are equipped with RFID tags and each tag is mapped to particular waste material. This approach helps in waste material classification for proper and efficient recycling and disposal.

From the study performed on the RFID enabled trash bin level monitoring systems, the following inferences are obtained:

- RFID-based systems are unable to provide continuous real-time monitoring of the bin’s filled level;
- Trash bins are monitored only when the truck is within the range of RFID tags;
- RFID tags are using to identify the bins uniquely and they do not provide any data on the filled levels of the bins;
- Additional infrastructure is required to obtain the filled levels of the trash bins.

## 4. Wireless Sensor Networks Based Solid Waste Management Systems

A Wireless Sensor Network (WSN) is a network of a large number of wireless sensors that are installed ad hoc to monitor the physical or environmental parameters of the system. Sensor nodes with an onboard CPU are used in WSN to monitor the environment in a specific area. The sensor nodes are connected to a central node called the coordinator node and all the coordinator nodes are connected to the base station, which serves as WSN’s processing unit. The base station is connected to the internet to share data. WSN already showcased its applications in various domains such as home automation, environmental monitoring, agriculture, industry, waste management, health, and fitness monitoring. Among the aforementioned applications, WSN presents a key role in designing and deploying real-time solid waste management systems. Some of the main characteristics of WSN based monitoring systems are as follows:

- Capacity for dealing with node failures;
- Optimal for nodes equipped with batteries;
- Nodes mobility and heterogeneity;
- Scalability to a vast distribution scale.

### 4.1. Network Architecture

**Figure 5:**
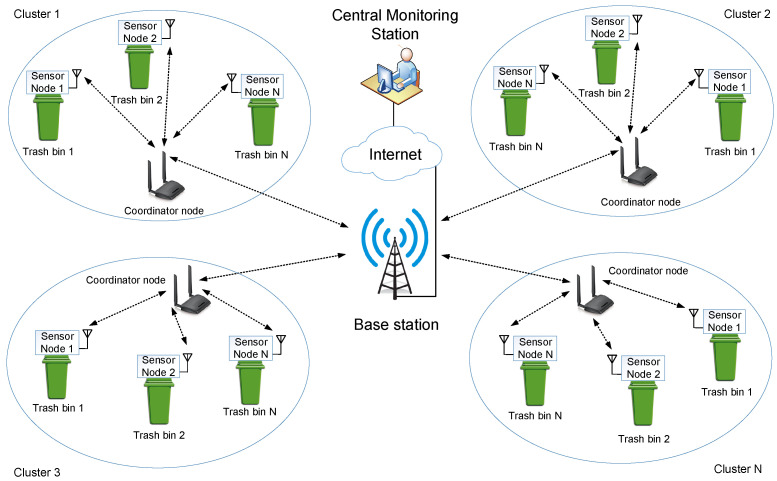
A typical WSN architecture for solid waste handling systems.

A typical WSN architecture for solid waste handling system is shown in Figure 5. The architecture mainly consists of clusters, base station, and central monitoring station. Each cluster includes several trash bins equipped with sensor nodes, and all these sensor nodes are connected to a coordinator node. The coordinator nodes of various clusters are connected to the base station, which will act as WSN’s processing unit. Furthermore, the base station will share data to the internet for establishing remote monitoring facilities. A comparison of various wireless communication technologies that enabled WSN is shown in Table 2.

#### WSN-Based Trash Bin Level Monitoring Systems

The advancements in wireless technologies face-lifted solid waste management systems that were bound to the limitations of establishing real-time monitoring and wide-range deployments. This section presents a comparative analysis of various WSN-enabled trash bin level monitoring systems. Table 3 shows the summary of WSN-based systems concerning communication technologies. A WSN architecture for solid waste management based on Zigbee networking protocol is presented in [34]. The architecture comprises three modules: data collecting, data processing, and notification. The end nodes mounted on the trash bins measure the unfilled level and update the server through a Zigbee coordinator. Moreover, to save time and money, notifications are sent to the garbage pickup unit using the Telegram messaging service. An intelligent trash bin monitoring system with Zigbee network architecture is presented in [35]. The smart nodes enabled with the Zigbee networking protocol transmit the sensed data (filled level of trash bin) to the Zigbee coordinator. The Zigbee coordinator node receives data from all smart nodes and send data to the cloud server when sensed data crosses the threshold level. The cloud server will assess the degree of fullness of trash bins for optimal route planning for waste collection. In addition, the sensor nodes are designed for harvesting energy by using solar energy to extend the lifetime of the nodes. However, the system fails to provide a real-time visualization of the trash bin’s filled level.

A network architecture employed with ArgosD sensor nodes is presented in [36]. The nodes are enabled with CC2420 RF transceivers, MSP430F1611 microprocessor, and a sensor. The graphical user interface displays the bin level but not the organizational level. In addition, as compared to conventional approaches, the current consumption and wake-up speed of the devices used are high.The modeling of a wireless sensor network to monitor the unfilled level of bins through a central monitoring station is presented in [37]. The wireless monitoring device comprises an ultrasonic sensor, MSP430F2274 microcontroller, and a CC2500 radio that uses the simpliciTI network protocol. The sensor nodes that run on battery power would last around 288 days for a 10,400 mAh power bank. Moreover, to send unfilled data to the remote monitoring station, this model requires a personal computer or a personal digital assistant that increases the overall cost of the system.

The design and development of a wireless sensor network powered by a solar energy harvesting system to monitor the unfilled level of the trash bin is presented in [38]. Each bin has a node called Solar Powered Wireless Monitoring Unit (SPWMU), which contains a sensor that measures the unfilled level of the bins and transmits data to the Solar Powered Wireless Access Point Unit (SPWAPU). Experiments have been carried out to verify the proposed system. The battery charging time and life expectancy of the SPWMU were calculated, and the average charging time was determined to be 6.26 h, with the charge lasting 27 days and 17 h. Even on rainy days, the unfilled level of bins can be monitored perfectly without interruption. Furthermore, this system does not require additional PC or PDA for sending data to the monitoring station, which ultimately reduces the effective deployment cost of the system.

A self-powered, simply connected WSN-based solid waste management system is presented in [39]. The sensor nodes attached to the trash bins measure the unfilled level of the bins and send it to the wireless access point. The data received from several sensor nodes will be forwarded to the central monitoring station for analysis and visualization. The nodes are equipped with solar panels for energy harvesting. Significant experiments were carried out to validate the system in terms of sensor accuracy, the lifetime of the node, and the maximum range between nodes and the wireless access point. Moreover, the Graphical User Interface (GUI) is designed with progressive bars to represent the dynamic unfilled levels of the trash bins.

## 5. IoT-Enabled Solid Waste Management Systems

IoT has emerged as one of the most crucial technologies of the twenty-first century in recent years [40]. The advances in technologies such as low power sensors, long-range connectivity, cloud computing, and machine learning made the practical establishment of IoT systems faster. An IoT environment consists of a network of web-centric smart devices that use embedded electronic devices such as communication hardware, sensors, and CPUs to collect, send, and process the data from their surroundings. Connecting IoT devices to any edge device such as an IoT gateway allows the sharing of sensor data that can be sent to the cloud for examination. Sometimes, these devices interact among themselves and carry out action on the data they receive. Despite the fact that the users can interact with devices such as setting them up, providing instructions, and extracting data, a greater part of the work is handled by IoT devices alone, eliminating human intervention.

IoT already showcased its robustness and efficiency in various domain-specific applications especially in wearables, smart home, healthcare, smart cities, agriculture, industrial automation, etc. The solid waste management system is one of the most important IoT-based services provided in smart cities. The main features of IoT-enabled solid waste management systems are as follows:

- No missed pickups: The data recorded from the smart bins assist in reducing missed pickups. The authorities will be automatically notified if the sensors detect that the garbage container is full. Then, the IoT waste management system enables the scheduling of next pickup for this location. This simplifies the process of waste management and reduces overflowing garbage cans.
- Waste production analysis: Throughout the day, the connected devices keep track of how quickly the bins fill up and how often they empty in different locations. The analysis of these data opens the possibilities of better trash bin distribution, elimination of improper disposal techniques, and even waste reduction at the landfill.
- Route Optimization: The real-time data provided by the smart trash bins can be used to determine the best paths for garbage collection by prioritizing the most required regions.

### 5.1. Network Architecture

**Figure 6:**
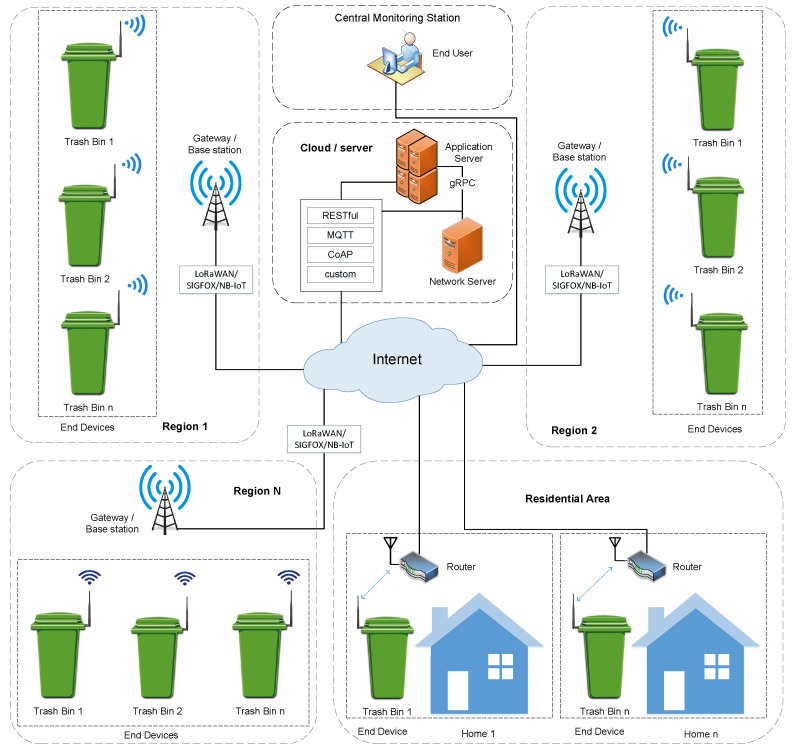
A generic IoT architecture for solid waste handling system.

A generic IoT architecture for solid waste handling system is shown in Figure 6. The architecture comprises four sections namely IoT end device, gateways or base station, cloud server, and end-user.

- IoT end device: It consists of sensors to measure the unfilled level or weight of the trash bins, a microcontroller to perform local processing, and a radio device to establish wireless connectivity with gateways or base stations.
- Gateway or Base stations: Several communication technologies are available to establish connectivity between the end devices and the cloud server. According to the adopted wireless technology, the gateway or base station will act as a bridge between the end devices and the cloud server. For instance, a gateway for LoRaWAN devices, a base station for Sigfox and NB IoT devices, and a wireless router for Wi-Fi devices.
- Cloud Server: For IoT applications, cloud servers are preferred due to their flexibility, scalability, and secure authentication process.
- End-user: Remotely monitoring a system can be performed by the end-user. The end-user may be an employee of the solid waste management company or a person in charge of the solid waste collection department in a municipality or corporation. A hierarchical view of trash bins’ filled level can be obtained through a web page or application software.

Furthermore, automated route optimization for waste pickup trucks is another part of waste management operations. To collect trash, these trucks usually follow a certain path every day. Drivers are often unaware of how full a trash bin is filled until they come across it. IoT solutions in waste management are helping to improve this situation by providing truck drivers with real-time information of the actual fill level of trash bins.

#### IoT Embedded Trash Bin Level Monitoring Systems

IoT-embedded trash bin level monitoring systems are the latest approach towards building smart cities. A wide range of IoT-based systems, starting from general trash bin monitoring to waste sorting and recycling is available in the literature. This section provides a comparative analysis of various IoTp-embedded trash bin level monitoring systems. Table 4 shows the summary of IoT-based solid waste handling systems.

An approach for the optimized handling of solid wastes in urban areas is proposed in [41]. A combination of proximity and weight sensors is incorporated to obtain the status of trash bins (i.e., weight/filled level). A Raspberry Pi microcomputer is acting as the gateway to transmit the measured data to the smart-M3 platform. However, this system requires additional gateways to increase its wireless range. Cloud-based smart waste management for waste collection and recycling is proposed in [42]. This system consists of separate trash bins for different types of wastes such as organic, plastic, bottles, etc. However, the study has not specified the details of the sensors and wireless technology adopted for deployment. In [43], the author describes various waste disposal methods in which IoT-based systems can be implemented. A smart box that is powered with solar energy is proposed in this system for bin level measurement. However, this work fails to present the architecture in detail. A system for screening and sorting plastic resin is proposed in [44]. After removing plastic wastes, the remaining wastes are used for biogas plants. In [45], the author describes a smart bin system that uses a mesh network to monitor the degree of fullness of the trash bin. The network is deployed using low-power radio at the 2.4 GHz band. The collected data are sent to a remote server for the analysis and waste collection route optimization. A smart garbage bin for streets is presented in [46]. This system sends the sensor ID and geolocation of the almost-filled bins to authorized persons. However, the design of the nodes and other technical details were not presented clearly. In [47], an IR sensor-based smart waste management system using IoT is presented. The trash bins’ filled levels are sent to an Intel Galileo Gen 2 board through RF modules. This work also presented a comparison with some of the existing systems.

Most of the systems discussed here use short-range communication technologies such as Wi-Fi, Zigbee, RF, etc. These short-range communication technologies require repeaters to increase their wireless range and are not suited for the low power consumption nature of IoT nodes. The evolution of low power wide area networks (LPWAN) addresses the above limitations by introducing communication technologies that are capable of transmitting data in long-range with low power consumption. Table 5 shows the features of the most popular LPWAN protocols, such as LoRaWAN, Sigfox, and NB-IoT. The LoRa Alliance maintains LoRaWAN, which is an open specification that primarily serves as a network layer protocol for handling communication between LPWAN gateways and end-node devices [48]. Sigfox [49] is a software-based communication system that manages all networks and computes complexity in the cloud rather than on the devices themselves. When all of this is taken into account, connected devices’ energy consumption and prices are reduced considerably. The Third Generation Partnership Project (3GPP), which is responsible for the standardization of cellular systems, has launched NB-IoT [50] to address the needs of very low data rate devices that must connect to mobile networks and are often powered by batteries.

Among the aforementioned LPWAN technologies, LoRaWAN is preferred for the deployment of IoT-based trash bin level monitoring systems because of the following features:

- LoRaWAN is an open specification, whereas NB-IoT and Sigfox are proprietary network protocols.
- LoRaWAN allows the establishment of private networks in which sensor nodes, gateways, and backhaul can be deployed by the user for a specific application. This is in contrast to NB-IoT and Sigfox, where a user needs to pay for connecting their sensor to the networks.
- LoRaWAN supports firmware upgrade over the air (FUOTA) that enables the remote firmware update of the multiple devices deployed over the network.
- LoRaWAN supports the Adaptive Data Rate mechanism for the optimization of power consumption, airtime, and data rates, whereas NB-IoT and Sigfox do not support this feature.

Table 6 shows the summary of the state-of-art, LoRa-enabled. IoT-based trash bin level monitoring systems. Automated waste management systems using sensors to monitor bin state, TensorFlow-based object detection for garbage identification and categorization, and the LoRa communication protocol for long-range, low-power data transmission were presented in [51]. Due to its lightweight nature, the pre-trained object identification model SSDMobilnetV2 can operate well with Raspberry Pi 3 Model B+. The model could detect and classify waste into different categories such as paper, plastic, and metal. Nevertheless, the accuracy of the model can be enhanced by increasing the frequency of training data, the frequency of captured images of waste, and the training period. The work in [52] discusses the suitability of waterproof ultrasonic sensors for measuring the level of waste within a trash bin. The proposed sensing node has been evaluated in the laboratory, demonstrating its efficacy. The value of the waste level inside the bin is determined with a precision of 2–3 cm; this value is sufficient since waste is not spread uniformly inside the bin. Following this initial testing, a network comprising five sensor nodes coupled to a single-channel LoRa gateway is constructed and implemented in real-time. The implementation of an Internet of Things (IoT) architecture for waste management optimization in the context of Smart Cities is presented in [53]. This work describes a novel sensor node topology based on the utilization of low-cost and low-power components. All the sensor nodes are equipped with a single-chip microprocessor, an ultrasound-based sensor for measuring the fill level of trash bins, and a LoRa transmission module. A LoRaWAN network is established to evaluate the performance of the proposed system. Moreover, it evaluates the end node in detail by focusing on energy-saving technologies and strategies, intending to increase battery life by reducing power consumption via hardware and software optimization.

A waste monitoring and management system for rural areas is presented in [54]. A low-power wireless node prototype is built to determine the weight, filling level, and temperature of the trash bin. This form of monitoring enables the collection and analysis of progressive filling data for each trash bin, as well as the generation of notifications in the event of an incident. The system enables a module for determining the most efficient waste collection routes. This module constructs routes dynamically based on data acquired from deployed nodes, saving energy, time, and, ultimately money. In [55], an IoT system based on the LoRa network is deployed and evaluated for feasibility in the development of an economically viable digital waste management system for Industry 4.0 and smart cities. As identified in this work, the primary challenge to successful commercialization is the cost of the sensor accessories, which is essentially driven by the ultrasonic distance sensing portion, where dependable waterproof sensors suitable for outdoor circumstances are expensive to deploy via the LoRa network.

A LoRaWAN-based solid waste management system with an energy harvesting sensor node is presented in [56]. The real-time deployment of the proposed architecture is performed and various evaluation metrics are provided to prove the feasibility of the work. In addition, an intelligent user interface is developed to locate the bins according to the filled levels. An architecture for building and developing a customized sensor node and gateway based on LoRa technology for accurately determining the filled level of bins is presented in [57]. This work evaluated the architecture’s energy consumption by simulating it using the Framework for LoRa (FLoRa) simulation while adjusting several essential LoRa communication parameters. Moreover, it also provides the evaluation metrics for the LoRaWAN network such as long-range, data rate, ToA (time on-air), LoRa sensitivity, link budget, and battery life. By considering the practical compatibility issues of short-range and long-range communication protocols, a dual network architecture is proposed in [58]. This work chose a WiFi network for residential trash bin monitoring and LoRaWAN for public trash bin monitoring. Moreover, the custom-designed sensor nodes were evaluated in terms of power consumption and energy harvesting capabilities.

## 6. Comparison and Discussion

A comparative study is performed in this article to investigate how technological advancements in communication and sensor design revolutionize the solid waste management sector. The study has emphasized three major approaches via RFID, WSN, and IoT in solid waste management. This section presents an overall comparison of the aforementioned approaches on the basis of the study performed in this work.

RFID-based solid waste management systems are enabled with GPRS and GIS for remote monitoring and route planning respectively. The RFID tags shall be considered as one of the best methods for identifying trash bins uniquely. Moreover, these approaches are mostly truck-oriented, in which significant computing loads are on the infrastructure deployed on the truck. In [27,28], trash bin surveillance is performed only by using a camera equipped on the truck. The aforementioned systems did not use any sensors while the bin status is captured before and after waste collection with the camera-equipped on the trucks. These approaches are not completely automated since the truck driver is responsible for adjusting the camera manually for accurate image capturing. Furthermore, the adoption of GIS makes a perfect fit for route optimization and the allocation of new bins. However, the requirement of a larger database and heavy processing power for image storage and image processing, respectively, makes the deployment expensive. In most of the works, a GPS sensor is incorporated to obtain the geolocation of trash bins. Since trash bins are stationary, the GPS sensor can be avoided in the node design and the same functionality can be obtained by mapping the RFID of every trash bin to the geolocation. The possibilities of the ghost tags and proximity between the truck and the trash bins for accurate tag reading are some of the challenges in these approaches. Moreover, none of the RFID-based systems studied in this work were able to provide real-time surveillance of the trash bins.

WSN-based solid waste management systems shall be considered as a breakthrough in the waste management industry. In contrast to RFID-based systems, real-time surveillance of the trash bins is established through WSNs. The adoption of wireless technologies such as Zigbee, SimpliciTi, etc., has reduced the power consumption of the sensor nodes, which will ultimately result in the improved lifetime of the batteries. Meanwhile, Wi-Fi is identified as one of the preferred communication technologies in many works studied in this survey even though its power consumption is relatively high when compared with similar communication protocols. This may be due to the fact that Wi-Fi is a popular communication technology and people are used to this technology due to the usage of smart devices or gadgets. Moreover, energy harvesting methods are adopted to enable self-powering to the sensor node. The works presented in [35,38,39] designed sensor nodes equipped with solar panels to extract energy from sunlight to charge the batteries. Nevertheless, a complete design of the charging circuit for energy harvesting is not presented in the aforementioned works. Most WSN-based approaches considered in this study are designed with GUIs to provide the remote monitoring of trash bins. Particularly in [39], progressive bars are represented to indicate the real-time unfilled level of the trash bins. Most of the WSN based solid waste management systems require a personal computer to send data to remote servers. The wide-area deployment of the trash bins is possible only through repeaters and other associated additional infrastructures. Consequently, all these lead to the increment of the overall cost for system implementation and complex designs. Furthermore, these approaches lag interoperability with other communication protocols.

The technological advances in sensor design and lightweight communication protocols pave the way for another trend in solid waste management. IoT-based solid waste management approaches are superior to previous approaches in terms of interoperability and the self-configuring capability of the monitoring nodes. Several IoT-based approaches are analyzed in this study and many of them are presenting promising design and experimental proofs for the feasibility of the proposed systems. The industry also showing favor toward IoT-based systems through their new releases of low-power microcontrollers integrated with radio devices and low power sensors. Consequently, it reduces the overall power consumption of the sensor nodes. A Wi-Fi and Zigbee-based solid waste management system enabled with IoT is discussed in [41,45], respectively. Even though the aforementioned approaches are feasible for real-time deployment, they are not a perfect solution when considering wide-area deployment for smart cities. Short-range communication protocols require additional infrastructure to increase their coverage and ultimately results in additional cost and complexity in deployment. The adoption of LPWAN protocols such as LoRaWAN, Sigfox, and NBIoT shall be considered as a solution to overcome the aforementioned limitations. LPWAN protocols are capable of sending data to long-range (several km) distances while consuming low power. From the study performed on LPWAN communication technologies, LoRaWAN shall be considered as the best choice for IoT-enabled solid waste management systems since they are open source and easy for distributed node deployment. Moreover, the low data rate of LoRaWAN is sufficient for sending bin status periodically. LoRaWAN-based solid waste management architecture is presented in [51,52,53], but the end nodes are designed with evaluation boards; consequently, the performance metrics are not true to industry standards. Meanwhile, in [54,55,56,57,58], customized nodes are designed and performance metrics are evaluated with real-time deployment.

An estimated cost comparison of some of the promising IoT-based approaches identified from the study is presented in Table 7. It can be inferred that the system developed with customized node designs is costlier than the others developed with evaluation boards or development boards. However, customized node designs would be superior in achieving low power consumption, an extended lifetime of batteries, and compactness.

The discussion on the state art solutions for waste management will not be completed without mentioning commercially available solutions. Some of the major commercial solid waste management systems in the industry are highlighted here. Bigbelly [59] is one of the leading providers of commercial smart garbage and recycling systems, with installations in cities, campuses, and workplaces in more than 50 countries worldwide. BIN-E [60] is an IoT-enabled trash bin that automatically sorts and compresses recyclables. To make waste management convenient and effective, it combines unique AI-based object recognition, fill level control, and data processing. EcoBins [61] is a smart city service that uses IoT technology to improve municipal waste collection operations. Sensor data are sent to the main server through a local GSM network for cloud-based analytics. Nordsense [62] provides another commercial approach for smart waste management to make cities clean, green, and smart. It offers a complete end-to-end solution based on IoT technology and an in-house developed navigation application. However, on the basis of the study performed on the state-of-art approaches and by considering commercially available solutions towards solid waste handling, there are several factors that need to be considered for the design of heterogeneous systems for addressing solid waste handling needs in urban areas.

## 7. Research Gaps and Future Direction

As far as waste management is concerned, it is a series of actions taken from the root to the final disposal of waste materials. The evolution of communication technologies and advancements in sensor designs revolutionized the conventional waste management system into an intelligent system. From the study performed on the various solid waste handling systems for smart cities, many research works present promising architecture and network models, which can be considered for real-time deployment. Nevertheless, this work identified some of the research gaps that need to be addressed for enhancing solid waste management in smart cities. This section describes various research gaps identified from the literature and the respective future directions to address the same.

- Self-powered end nodes: IoT systems comprise a large number of end nodes and gateways or base stations; while the end nodes are deployed at various locations, the gateways or base stations gather the data from the end nodes and send it to the internet for further analysis. The end nodes are preferably powered with batteries, and the frequent replacement of batteries is impractical when considering the wide range deployment of nodes. Some works in the literature proposed energy harvesting by using solar panels to address this issue. Still, there is a large scope of systems that need to adopt advanced methodologies to enhance the design of IoT-enabled solid waste handling systems with self-powered end nodes:
- Edge computing enabled waste segregation: Currently, waste segregation is implemented by placing multi-colored or labeled bins for the easy classification of the waste material. Even if it is an easy method of collecting a particular kind of waste, this approach cannot always be successful because, by mistake, people may place waste in the wrong bins and it will disturb proper segregation. Nevertheless, waste segregation by the bin itself would be a solution. There is a solid research scope in the aforementioned scenario to design edge computing-enabled smart nodes incorporated with image processing techniques to implement waste segregation.
- Hybrid network architecture: Waste generation is dynamic and varies concerning the environment. For instance, the frequency of filling of a trash bin installed at public places will be dynamic and the filling of trash bins installed at houses or residential areas will be probably in a uniform manner. From the survey performed on existing approaches, it is evident that LPWAN communication technologies are best suited for monitoring bins at public places since bins are deployed at distant locations. Similarly, short-range communication technologies such as Wi-Fi are best suited for monitoring trash bins located at houses since most houses will be equipped with a Wi-Fi router for internet connectivity. Nevertheless, none of the existing systems describe solid waste management models or network architectures for waste management in flats or apartments. Moreover, waste management in urban areas is incomplete without addressing the aforementioned scenario. As a result, a lot of research attention is required in developing hybrid network architectures that are capable to address the solid waste management requirements in public areas and residential areas differently.
- Smart Transportation: Transportation plays a crucial role in an efficient waste management system. Most of the existing approaches send the trash bin status to the central monitoring station or cloud servers for performing data analysis. On the basis of this analysis, waste collection routes will be scheduled and a truck or waste collection vehicle shall follow this scheduled path for optimized waste collection. Meanwhile, an unscheduled trash bin may become completely filled and miss the pick up. Thus, there is a need for dynamic path optimization centered on the waste collection vehicles’ live location and the real-time status of the trash bins.
- Customized node design: Based on the study performed on the various solid waste management systems, it is inferred that most of the end nodes are employed with development or evaluation boards. Consequently, the performance metrics obtained for those nodes will have some tolerance levels when compared with dedicated nodes designed for trash bin monitoring. Even though some works in the literature have designed customized nodes for compactness and power efficiency, there is still a scope for further enhancement. For instance, the majority of works considered in this study follow some hypothetical conditions, such as the waste-filled level being uniform and a single ultrasonic sensor can detect the unfilled level of the trash bins. However, in real scenarios, an end node with a single sensor will not be sufficient for detecting the unfilled level of larger trash bins. Therefore, there is a need for customized end nodes with multiple sensors for detecting the unfilled levels of larger trash bins efficiently.

## 8. Conclusions

A comprehensive study on various technological approaches towards solid waste management for smart cities is presented in this paper. The literature is categorized into three sections based on the evolution of technology. Firstly, RFID-based approaches for solid waste management have been discussed and analyzed with typical system architecture. The lack of real-time monitoring, short-range communication, and the need for additional infrastructure for trash bin monitoring are some of the challenges observed in this approach. Secondly, WSN-based approaches for solid waste management have been analyzed. These approaches are superior to RFID but face challenges in designing power-efficient and easily deployable systems. Finally, state-of-the-art IoT-enabled solid waste management systems have been studied. Low power sensors, wireless microcontrollers, and lightweight communication protocols enhanced IoT-based approaches toward solid waste management. Moreover, these approaches uplifted the WSN based systems with interoperability and dynamic adaptation of the nodes. On the basis of this survey, it is concluded that IoT-based solutions are optimal for the deployment of solid waste handling systems in urban areas. In addition, LoRaWAN is observed as the preferred communication protocol among LPWAN technologies that enabled IoT-based trash bin deployments in urban areas. Nevertheless, a lot of research scope is there for further improvement of these systems in ultra-low power node design and optimized energy harvesting methods for self-powered nodes. We envisioned that this comprehensive study on solid waste management systems emphasizing technology phases would be a good reference for future researchers to develop advanced systems for smart cities. Moreover, this would be a reference to the municipalities or waste management companies to plan the establishment of efficient solid waste handling systems in smart cities by adopting appropriate system models.

## Figures and Tables

**Table 1:** Summary of RFID-enabled Trash Bin Level Monitoring Systems.

Ref.	Bin Deployment	Sensors	Camera	GPS	GIS	Routing
[27]	exterior	none	Yes	Yes	Yes	undefined
[28]	exterior	none	Yes	Yes	Yes	undefined
[29]	not specified	infrared sensor	No	Yes	No	undefined
[30]	exterior	ultrasonic sensor, pressure sensor,	Yes	Yes	Yes	undefined
[31]	exterior	load cell sensor	No	No	No	undefined
[32]	interior/exterior	photoelectric, image sensor	No	Yes	No	defined
[33]	exterior	digital weight scale	No	No	No	undefined

**Table 2:** Comparison of popular WSN-enabled wireless technologies.

Wireless Technology	Wireless Range	Power Consumption	Operating Frequency	Data Rate
Zigbee	10–100 m	Low	2.4 GHz	20–250 kbps
Wi-Fi	100 m	Medium	2.4 GHz, 5 GHz	10–100 Mbps
Bluetooth LE	>100 m	Low	2.4 GHz	125 kbps–2 Mbps
Z-Wave	15–150 m	Low	sub-GHz	9.6–40 kbps
IEEE 802.15.4	10–20 m	Low	2.4 GHz	250 kbps
SimpliciTi	50 m	Low	2.4 GHz	250 kbps

**Table 3:** Summary of WSN-based trash bin level monitoring systems.

Ref.	Sensor	Microcontroller	Wireless Technology	Communication Network	GPS	Visualization	Energy Harvesting
[34]	Ultrasonic sensor	Arduino Uno	Zigbee	Mesh	Yes	No	No
[35]	Ultrasonic sensor	Arduino Pro Mini	Zigbee	Star	Yes	No	Yes
[36]	ArgosD sensor	MSP430F1611	IEEE 802.15.4	LoWPAN	No	Yes	No
[37]	Ultrasonic sensor	MSP430F2274	SimpliciTi	WLAN	No	Yes	No
[38]	Ultrasonic sensor	MSP430F2274	SimpliciTi	WLAN	No	Yes	Yes
[39]	Ultrasonic sensor	ATSAMW25H18	Wi-Fi	WLAN	No	Yes	Yes

**Table 4:** Summary of IoT based solid waste handling systems.

Ref.	Sensors	Radio Technology	Wireless Range	GPS	Energy Harvesting
[41]	weight sensor, proximity sensor	Zigbee	Short	No	No
[42]	level sensor	Not Specified	Not Specified	No	No
[43]	Not Specified	Wi-Fi	Short	No	Yes
[44]	Ultrasonic sensor, load cell	GSM	long	Yes	No
[45]	Ultrasonic sensor	Wi-Fi	short	Yes	No
[46]	Ultrasonic sensor	GSM	long	No	No
[47]	IR sensor	RF	Short	No	No

**Table 5:** Comparison of popular low-power wide-area network.

Communication Technology	Wireless Range	Power Consumption	Operating Frequency	Data Rate	Modulation Technique
LoRaWAN	<15 km	Low	Sub-GHz	0.3–50 kbps	SS Chirp
SigFox	50 km	Low	Sub-GHz	100 bps	DBPSK
NB-IoT	<35 km	Low	Cellular Bands	200 kbps	QPSK

**Table 6:** Summary of various LoRa-enabled IoT-based trash bin level monitoring systems.

Ref.	Sensor	Microcontroller	Radio Device (LoRa)	Custom Node Design	GPS	Energy Harvesting	Real Time Deployment
[51]	Camera, Ultrasonic Sensor	Arduino Uno	SX 1272	Yes	Yes	Yes	Yes
[52]	Ultrasonic Sensor	Atmega328P	SX 1272	Yes	No	No	Yes
[53]	Ultrasonic Sensor	Atmega328P	SX 1278	Yes	No	No	Yes
[54]	Ultrasonic Sensor, Load cell, Temperature sensor	ATSAML21	SX 1276	Yes	No	No	Not Specified
[55]	Ultrasonic Sensor	Raspberry Pi3	IP67 LoRa gateway	Yes	No	No	Not Specified
[56]	Ultrasonic Sensor	ATmega 2560	RN2903	Yes	Yes	Yes	Yes
[57]	Ultrasonic Sensor	Atmega328P	SX 1278	Yes	No	No	Yes
[58]	Ultrasonic Sensor	ATmega 2560	RN2903	Yes	Yes	Yes	Yes

**Table 7:** Estimated cost comparison of IoT-based approaches.

Ref.	Title of the Project	Estimated Prototype Cost in USD
[39]	An IoT-based bin level monitoring system for solid waste management	107
[51]	An internet of things based smart waste management system using LoRa and tensorflow deep learning model	180
[53]	A low power IoT sensor node architecture for waste management within smart cities context	57
[56]	A LoRaWAN IoT enabled Trash Bin Level Monitoring System	161

## Data Availability

Not applicable.
